# An Attempted Substitute Study of Total Skin Electron Therapy Technique by Using Helical Photon Tomotherapy with Helical Irradiation of the Total Skin Treatment: A Phantom Result

**DOI:** 10.1155/2013/108794

**Published:** 2013-07-31

**Authors:** Chi-Ta Lin, An-Cheng Shiau, Hui-Ju Tien, Hsin-Pei Yeh, Pei-Wei Shueng, Chen-Hsi Hsieh

**Affiliations:** ^1^Division of Radiation Oncology, Department of Radiology, Far Eastern Memorial Hospital, New Taipei City 220, Taiwan; ^2^Department of Biomedical Imaging and Radiological Science, China Medical University, Taichung 404, Taiwan; ^3^Department of Radiation Oncology, Tri-Service General Hospital, National Defense Medical Center, Taipei 114, Taiwan; ^4^Department of Medicine, School of Medicine, National Yang-Ming University, Taipei 112, Taiwan; ^5^Institute of Traditional Medicine, School of Medicine, National Yang-Ming University, Taipei 112, Taiwan

## Abstract

An anthropomorphic phantom was used to investigate a treatment technique and analyze the dose distributions for helical irradiation of the total skin (HITS) by helical tomotherapy (HT). Hypothetical bolus of thicknesses of 0, 10, and 15 mm was added around the phantom body to account for the dose homogeneity and setup uncertainty. A central core structure was assigned as a “complete block” to force the dose tangential delivery. HITS technique with prescribed dose (*D*_*p*_) of 36 Gy in 36 fractions was generated. The radiochromic EBT2 films were used for the dose measurements. The target region with 95.0% of the *D*_*p*_ received by more than 95% of the PTV was obtained. The calculated mean doses for the organs at risk (OARs) were 4.69, 3.10, 3.20, and 2.94 Gy for the lung, heart, liver, and kidneys, respectively. The measurement doses on a phantom surface for a plan with 10 mm hypothetical bolus and bolus thicknesses of 0, 1, 2, and 3 mm are 89.5%, 111.4%, 116.9%, and 117.7% of *D*_*p*_, respectively. HITS can provide an accurate and uniform treatment dose in the skin with limited doses to OARs and is safe to replace a total skin electron beam regimen.

## 1. Introduction

Radiation therapy achieves an effective treatment for cutaneous T-cell lymphoma affecting the superficial region [1]. This treatment delivers an adequate and uniform dose to the whole body superficial area. Historically, mycosis fungoides (MF) is treated mainly with total skin electron beam therapy (TSEBT). One of the most widely used TSEBT techniques was six dual fields [2]. Dosimetrically, TSEBT at energies of about 3–7 MeV at the surface of a standing patient may result in significant dose variations due to variable skin distance, self-shielding, irradiated fields overlapping, and patient motion [3, 4]. Special areas of the body, such as the perineum and eyelid [3] or inframammary fold [4], showed large deviations (up to 40%) from the prescription dose [3, 4]. Although the *in vivo* measurement of different treatment areas can realize the dose distribution in a certain region, a complicated distribution of underdosed areas can scarcely be treated with a homogeneous dose using supplemental patch fields.

Helical tomotherapy (HT) has advantages in irradiating extended volumes with treatment length of up to 160 cm, continuously in a helical pattern without the need for field junction [5]. Previous publications demonstrated it is feasible for total marrow irradiation (TMI) techniques by HT to replace total body irradiation [6] or practicing for multiple myloma patients [7]. Additionally, HT for total scalp irradiation has shown that the employment of directional and complete blocking on the inner structures can effectively force the tangential delivery of the beamlets to the planning target volume (PTV), which can limit the treatment depth successfully [8].

Here, an anthropomorphic phantom is used to investigate the helical irradiation of the total skin (HITS) technique by HT planning system and compares with the conventional TSEBT administrated using a conventional linear accelerator. The dosimetry analysis, the uncertainty of dose calculation, the surface, and superficial doses are evaluated.

## 2. Materials and Methods

### 2.1. Treatment Plan

**Figure 1:**
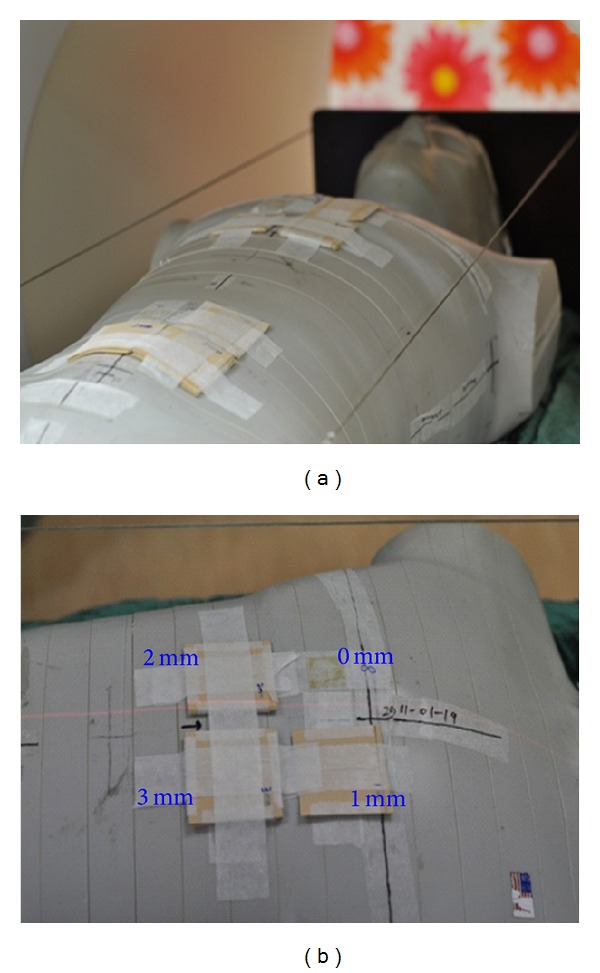
(a) Sheets of tissue equivalent material with thicknesses of 1, 2, and 3 mm were placed on the phantom surface as a bolus. (b) The EBT2 films were placed on the phantom surface and inserted between the bolus and phantom surface for dose measurements.

**Figure 2:**
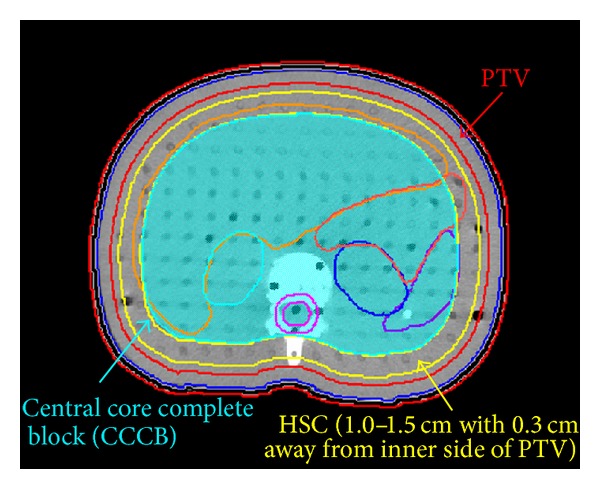
The phantom axial image displays the hypothetical target structure, the constraint object, and the central core structure.

A treatment planning CT was taken of an anthropomorphic body phantom (ATOM 701; CIRS, Norfolk, Va), placed in the supine position and immobilized using a Vac-Lok bag (CIVCO Medical Instruments, CO, Inc., Kalona, IA). Sheets of tissue equivalent material at thicknesses of 1, 2, and 3 mm were placed on the phantom surface as a bolus for dosimetry analysis (Figures 1(a) and 1(b)). The image set was transferred to the treatment planning system (Pinnacle3 Version7.6C) using a hypothetical target volume including 5 mm depth all around the body surface contoured as the clinical target volume (CTV). The inner side of CTV plus 1.5 cm and the outer side of CTV added 0.5 cm, these areas were defined as PTV. From the shoulder to wrist and legs above ankles that areas under the skin with 0.5 cm were defined as the CTV_extremity_. Hands and feet were contoured without bone sparing as CTV_hand_ and CTV_feet_. The PTV_extremities_ were defined as the volume with two dimensional expansion of 1.0 cm from the CTV_extremity_, CTV_hand_, and CTV_feet_. A hypothetical constraint structure (HCS), 0.3 cm margin away from the inner side of PTV with 1.0–1.5 cm 2 dimensional expansion, was contoured for the dose constraint. The organs and tissues that adjacent to the inner side of HCS were contoured together as central core complete block (CCCB) and used to restrict the photon beams to be obliquely incidence for increasing the superficial dose and reducing the internal organ dose. (Figure 2) Lung, heart, liver, kidney, spleen, intestine, ovary, stomach, and spinal cord were contoured as organs at risk (OARs). Additional margins of 0, 10, and 15 mm were extended from the phantom surface individually for a different plan contoured as a hypothetical bolus to account for the dose homogeneity increase at the superficial region and setup uncertainty. The CT images and structures were then transferred to the Tomotherapy Hi-ART planning system (v. 3.2.2.35. TomoTherapy Inc., Madison, WI). A plan with prescription dose (*D*_*p*_) of 36 Gy in 36 fractions to 95% of the PTV and the maximum dose less than 120% of the *D*_*p*_ was generated. The maximum irradiation length and width for HT were 160 and 85 cm, respectively. The slice thickness, pitch, and modulation factor parameters were assigned 2.5 cm, 0.287 cm, and 3.5, respectively. The dose constraints to the OARs and the HCS were adjusted accordingly during optimization to achieve a plan with a rapid dose distribution falloff. For all measurements a predelivery megavoltage CT (MVCT) scan was taken for position alignment. 

### 2.2. Dose Measurement

Radiochromic EBT2 film with high spatial resolution and thin configuration (thickness of 0.234 mm and effective measurement depth of 0.153 mm in a layer) has been proven a viable tool for external beam dosimetry in the superficial region [9, 10]. All of the radiochromic EBT2 films used in this study were from the same lot number (International Specialty Products, Inc. Wayne, NJ). Each film sheet of 25 × 20 cm was cut into smaller pieces (size of 5 × 5 cm) for calibration and measurement. An Epson Perfection V700 flatbed scanner (Epson Seiko Corporation, Epson Seiko Corporation, Nagano, Japan) was used to scan all of the films at least 24 hours after film exposure. Films were scanned at a central scanner location and with the same orientation. The settings used were 48 bit color and 150 dpi (0.017 cm per pixel). The red channel data with 16 bit digital information were extracted and processed using the public domain software ImageJ Version 1.43 (National Institute of Health, Bethesda, MD, http://rsb.info.nih.gov/ij/). Calibration was performed by irradiating the each calibration film individually in a plastic water phantom perpendicularly to a 6 MV beam at dose levels from 0 to 300 cGy. The calibration curve was fitted using a polynomial function with the pixel value (PV) for each measurement film converted to dose accordingly.

To evaluate the buildup range of the doses on the surface and superficial regions for HITS, measurements were performed with EBT2 films placed on the phantom surface at different areas with boluses of different thicknesses of 0, 1, 2, and 3 mm added onto the films for dosimetry analysis. For clinical HITS application purposes increasing the surface dose and decreasing the unwanted air gaps under the dose buildup material, a custom-made neoprene diving suit was considered as the dose buildup material with the effective thickness relative to water evaluated. To account for the diving suit bolus effect, a piece of diving suit 3 mm thick was irradiated to a 6 MV beam to evaluate the effective thickness relative to water. To verify the calculated doses on critical organs, measurements were performed with EBT2 films inserted into the phantom at the critical organ locations.

### 2.3. Verification of Angular Dependence with EBT2 Film

**Figure 3:**
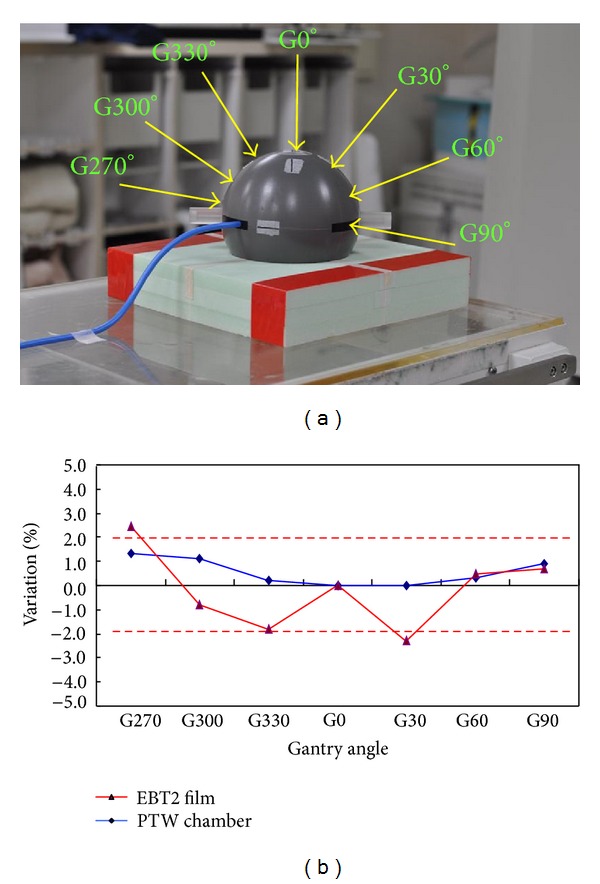
(a) PTW 31010 semiflex ionization chamber (PTW-Freiburg, Germany) as a standard without angular dependence. PTW chamber has been irradiated in spherical phantom to determine the ionization along the central axis to various gantry angles. (b) Angular dependence, measurements using EBT2 film and PTW semiflexible chamber.

We used spherical polystyrene phantom and PTW 31010 semiflex ionization chamber (PTW-Freiburg, Germany) as a standard without angular dependence. Reference condition was performed in 100 cm source axial distance (SAD), with field size 5 × 5 cm^2^ and using 6 MV photon beam 200 MU delivery. PTW chamber has been irradiated in a spherical phantom to determine the ionization along the central axis to various gantry angles. (Figure 3(a)) The ionization readings of each angle were normalized by the measurement value of normal incidence exposure. Each angle variation results will be recorded and analyzed. EBT2 film sheets have been irradiation in spherical phantom at the same position. Reference condition was performed in SAD 100 cm, with field size 5 × 5 cm^2^ and using 6 MV photon beam 250 MU delivery. An Epson perfection V750 PRO flat bed scanner (Epson Seiko Corporation, Epson Seiko Corporation, Nagano, Japan) was used to scan all the films at least 24 hours after film exposure. The optical density measurement position of EBT2 film was extremely small and identical to chamber exposure position. The variation of PTW ionization chamber results was compared with the EBT2. 

## 3. Results

### 3.1. Angle Effect and Uncertainty in Detail

The angular dependence of EBT2 film was small and applicable to clinical surface dose measurement. In the current study, the total uncertainty was less than 2.5% (Figure 3(b)).

### 3.2. Treatment Plan

**Figure 4:**
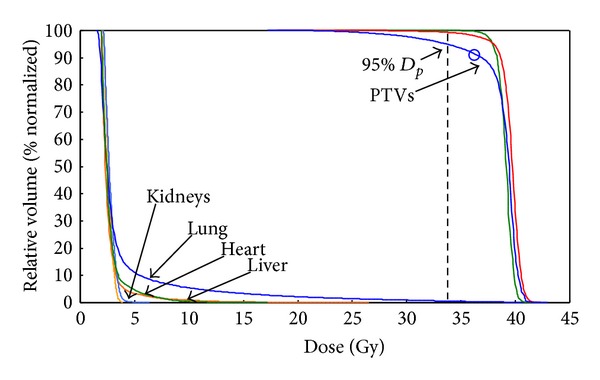
Dose volume histogram of helical irradiation of the total skin (HITS) with prescribed dose of 36 Gy in 36 fractions for treatment in a study phantom.

**Figure 5:**
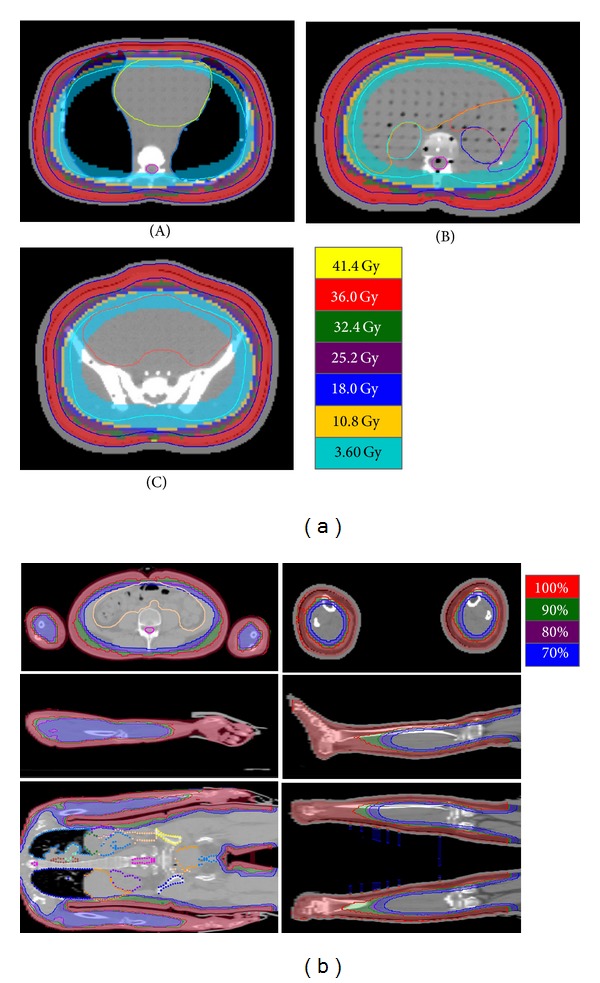
(a) Dose distributions at chest (A), abdomen (B), and pelvic (C) areas for helical irradiation of the total skin (HITS) treatment in a study phantom. (b) Due to our RANDO phantom without the thighs and arms, the CT images of whole body of one patient who received total marrow irradiation were used to replan with HITS technique to show the workable of dose delivery to extremities.

A homogenous dose in the target region was obtained. Ninety-five percent of the *D*_*p*_ was received by more than 95% of the PTV and the maximum dose was less than 116% of the *D*_*p*_. (Figure 4) The calculation mean doses for the critical organs were 4.69, 3.10, 3.20, and 2.94 Gy for the lungs, heart, liver, and kidneys, respectively. The HITS plan statistics with 10 mm hypothetical bolus are shown in Table 1. The calculated dose distributions are shown in Figure 5(a). The dose delivery duration was within 45 minutes. 

### 3.3. Dose Measurement

The dose differences between measurements and calculations for OARs were less than 0.8 Gy. In addition, the mean doses for OARs were between 2.9 and 9.1 Gy (Table 1). The dose of the vertex was 108.0% ± 1.2%. Due to the lack of arms and thighs for RANDO phantom, the skin dose of arms and thighs could not be measured from RANDO phantom. Therefore, the CT images of whole body of one patient who received total marrow irradiation were used to replan with HITS technique to show the workable of dose delivery to extremities (Figure 5(b)). For the TMI and HITS plans, the hand and feet were all irradiated without sparing; therefore the results of surface dose checked by EBT2 film could be similar. And the hands and plantar skin dose measured by EBT2 film of the TMI plan were 117.2% ± 2.8% and 108.6% ± 4.8%, respectively. The measurement doses on the phantom surface for plans with different hypothetical bolus thicknesses and the actual bolus were shown in Table 2. A higher superficial dose was obtained as a thicker hypothetical bolus was used. The effective thickness relative to water of the diving suit is 0.87 mm.

## 4. Discussion

Most TSEBT procedures are time consuming. Since patients requiring TSEBT are often elderly and weak, a long treatment time for a patient in a standing position is difficult to hold at a correct position and to ensure their safety. HITS sets patient in the supine position and immobilizes the patient with a vacuum bag, which is more stable and comfortable for a long duration treatment. Additionally, the requirement of room size for TSEBT is about 4 meters, which may restrict using the TSEBT technique. HITS do not need a large treatment room and can be performed without room size restriction. 

HITS employ a CCCB to force the majority of the beamlets to be delivered to the PTV tangentially. This limits the depth of the dose distribution and also makes the treatment more vulnerable to setup and respiratory motion errors. Using CCCB technique in HITS study, the mean doses for OARs, such as the lungs, heart, liver, intestine, and kidneys, were 4.7, 3.1, 3.2, 3.6, and 2.9 Gy, respectively (Table 1). Historically, whole heart doses up to 30 Gy were reasonably well tolerated [11]. In addition, limiting mean lung dose to ≤20–23 Gy can limit the risk of radiation pneumonitis to ≤20% in definitively treated patients with nonsmall-cell lung cancer [12]. Furthermore, Dawson et al. [13] reported when the mean liver dose less than 31 Gy (biologic effective dose = 30 Gy10 in 2 Gy/fraction) that was no cases of subsequent radiation-induced liver disease. Emami et al. [14] and Robert Cassady [15] suggested a total dose associated with a 5% and 50% risk of kidney injury at 5 years of 18–23 Gy and 28 Gy, in 0.5–1.25 Gy/fraction, respectively. With doses on the order of 50 Gy, late small-bowel obstruction or perforation rates of 2% to 9% had been observed after partial organ irradiation [16], concordant with the Emami et al. TD5/5 estimate [14]. According to the previous reports, HITS technique could provide safety for total skin irradiation.

 Hypothetical bolus is a method to overcome setup and respiratory motion errors but the dosimetric condition is different between the planned and delivered beams. An increment in the effective hypothetical bolus thickness in a steeply inclined incidence while optimization will make the dose distributions with a significant increment in the shallow region while treatment. This increment increases with the thickness of the hypothetical bolus (Table 2). Based on this study, the measurement doses on a phantom surface for a plan with 10 mm hypothetical bolus and bolus thicknesses of 0, 1, 2, and 3 mm are 89.5%, 111.4%, 116.9%, and 117.7% of *D*_*p*_, respectively. Ten mm of hypothetical bolus thickness will increase 10% of *D*_*p*_ to the superficial regions. Tomotherapy system delivers dose with 6 MV; the attenuation factor of this energy is about 4.5%/cm (0.45%/mm). Dosimetrically, attached boluses with thicknesses of 1, 2, and 3 mm on phantom surface will mainly affect the doses on surface. The differences of dosimetric effects caused by the boluses from the beam entrances away from the measurement regions will be less than 1.0%. In addition, the size of the bolus is about 1.5 cm larger than the measurement film in each side, and the film reading is accounted on the central area (about 3 × 3 cm). So, the scatter radiation can be accounted mostly in the measurement condition stated in this study. Therefore, the dose differences caused by the boluses in different thicknesses are sufficient to account for. Additionally, an appropriate hypothetical bolus thickness should be chosen according to the variations in surface positions of interfraction or intrafraction motion.

Gafchromic EBT2 film has high spatial resolution (thickness of 0.234 mm and effective measurement depth of 0.153 mm in a layer) [17], low energy dependency [18], and near tissue equivalent density (*Z*_eff_ = 6.84 for EBT2, *Z*_eff_ = 7.42 for tissue) [19]. The weak energy dependence of the EBT2 makes it most suitable for clinical use compared with other films [19]. The total uncertainty in the surface dosimetry using EBT2 film reported by Nakano et al., Hartmann et al. [20], and Richley et al. [21] are approximately 3.3%, 3.7%, and 5.5%, respectively. In the current study, the total uncertainty less than 2.5% competes with previous studies with better results (Figure 3(b)).

Consensus guidelines for delivery of TSEBT have been published by the European Organization for Research and Treatment of Cancer (EORTC) [22]. The EORTC recommends a total dose of 31 to 36 Gy prescribed to the skin surface to produce a dose of at least 26 Gy at a depth of 4 mm in the truncal skin along the central axis [22]. In our calculation, a surface dose is inadequate for HITS. To overcome this problem, a custom-made neoprene diving suit of 3 mm thickness that fits the patient's body curvature well is considered as the dose buildup material to increase the surface dose and to decrease the unwanted air gaps. The effective thickness relative to water of the diving suit is 0.87 mm, which is enough for dose buildup for HITS with 10 mm hypothetical bolus to achieve more than 110% of *D*_*p*_ on the skin surface (Table 2). 

HITS using 6 MV photon beams generates a plan with higher internal organ dose than the TSEBT technique. A mean dose of about 3.5 Gy (9.7 cGy/fx) is received by the OARs under HITS with 36 Gy in 36 fractions. The internal TSEBT dose is contributed mainly by the contaminated X-ray, typically ranging l%–4% (1–4 cGy/fx) of the maximum electron dose received at the surface [23]. Recent reports are interesting in revisiting the effectiveness of lower dose TSEBT in the management of MF [1, 24]. Based on Harrison's report [1], the overall response rates associated with low-dose TSEBT in the 10 to <20 Gy and 20 to <30 Gy ranges are comparable to those of the standard dose (≥30 Gy). The internal organ doses from HITS might be tolerable for a 36 Gy regimen and are safer for revisiting a low-dose TSEBT regimen. 

The drawback of HITS is the longer beam on time and probably the higher inner doses. Increased field width to 5.0 cm may shorten the duration significantly but the PTV coverage may reduce slightly or the maximum dose to PTV may increase by 10%–15%. 

## 5. Conclusion

To the best of our knowledge, this is the first phantom study to prove the possibility to replace TSEBT by HT with HITS technique. HITS technique provides an accurate and uniform treatment dose to the skin area in this phantom study. The internal organ doses were effectively spared using a tangential beamlets delivery method. A diving suit of 3 mm thickness for clinical purpose is needed to increase the surface dose and to decrease the unwanted air gaps.

## Figures and Tables

**Table 1:** The helical irradiation of the total skin (HITS) technique statistics with 10 mm hypothetical bolus for total skin and organs at risk (OARs).

	*D*_max⁡_ (Gy)	*V*_95%_ (%)	*D*_mean-*C*_ (Gy)	*D*_mean-*M*_ (Gy)
PTV	41.88	96.0	38.83	—
Heart	28.67	—	3.10	3.25
Lung	40.96	—	4.69	3.93
Liver	25.16	—	3.20	—
Kidney	4.38	—	2.94	3.16
Spleen	6.03	—	3.11	—
Intestine	25.80	—	3.40	—
Rectum	14.83	—	9.06	—

*D*_max⁡_ (Gy): maximum dose.

*V*_95%_ (%): target volume in % encompassed by 95% of the *D*_*p*_.

*D*_mean-*C*_ (Gy): TPS calculation mean dose.

*D*_mean-*M*_ (Gy): measurement mean dose.

**Table 2:** Measurement doses (% of *D*_*p*_) of the surface and superficial regions for the helical irradiation of the total skin (HITS) technique with different hypothetic bolus thicknesses.

	Hypothetic bolus thickness		
Depth (mm)	0 mm	10 mm	15 mm
Surface	73.2	89.5	86.4
1.0	91.4	111.4	118.5
2.0	101.7	116.6	118.9
3.0	101.9	117.7	120.9
